# Supplementary material for: Different honesty conceptions align across US politicians' tweets and public replies
Source: Nat Commun. 2025 Feb 6;16:1409. doi: 10.1038/s41467-025-56753-6 (PMC11802837; doi:10.1038/s41467-025-56753-6)
Supplement: Supplementary file 1 — Supplementary Information [file 41467_2025_56753_MOESM1_ESM.pdf]

# Supplementary Information: Different Honesty Conceptions Align Across US Politicians' Tweets and Public Replies

Fabio Carrella[1], Segun T. Aroyehun[2], Jana Lasser[3,4], Almog Simchon[5], David Garcia[2,4], Stephan Lewandowsky[1,6]

[1] School of Psychological Science, University of Bristol, Bristol, UK.
[2] Department of Politics and Public Administration, University of Konstanz, Konstanz, Germany.
[3] IDea_Lab, University of Graz, Graz, Austria.
[4]Complexity Science Hub, Vienna, Austria.
[5]Department of Psychology, Ben-Gurion University of the Negev, Beer Sheva, Israel.
[6]Department of Psychology, University of Potsdam, Potsdam, Germany.

Corresponding author(s): fabio.carrella@bristol.ac.uk;

## Supplementary Note 1

In our main analysis, we explore the alignment of two honesty framings—belief-speaking and fact-speaking—across political tweets (i.e., "seeds") and the replies they received. We describe these two honesty components as follows: belief-speaking prioritizes the genuine and authentic expression of beliefs, focusing more on a person's emotional or mental state rather than the objective state of the world, whereas fact-speaking emphasizes the accuracy of a statement and aims to convey the true state of affairs. This description implies a relationship between belief-based expressions of honesty and emotions.

We controlled for a particular emotional aspects of language, namely affective polarization, in the main analysis and discovered a contagion effect, where

the honesty component in the replies aligns with that present in the seeds, regardless of the political affiliations of the politicians and the repliers.

Here, we check the robustness of this result by controlling for positive and negative emotions in the seeds. We measured these two aspects using the Linguistic Inquiry and Word Count (LIWC) program [1]. LIWC is a text processing software that has been continuously developed for more than two decades and computes several indicator variables from text based on word lists generated by psychologists and validated in various experiments. LIWC analyzes texts to determine if words are included in predefined categories (e.g., negative and positive emotions, social ties, etc.); if so, the values associated with the matched categories increase. Unlike the Distributed Dictionary Representation approach [2], where textual scores represent the cosine similarity with the averaged embedded representation of a reference dictionary, LIWC categories are expressed as percentages of words in a document associated with a category.

We added two further standardized predictors, LIWC_pos and LIWC_neg, to the regression formula used in the main article to detect the contagion effect, thus obtaining the following equation:

$$\begin{aligned} \mathrm{FmB}_r \sim\ & \mathrm{FmB}_s \times \mathrm{I}_{score} \times \mathrm{Party} + \mathrm{Pol}_s + \mathrm{Date} \\ & + \mathrm{LIWC}_{pos} + \mathrm{LIWC}_{neg} + \mathrm{Pos} \\ & + (1|\mathrm{AuthorID/SeedID}) + (1|\mathrm{Topic}) \end{aligned} \tag{1}$$

Results, reported in the Supplementary Table 1, show that the correlation between the honesty conception of the seeds and that of the replies remains positive and significant ($t(97,510) = 19.396$, $p < 0.001$, $\beta = 0.075$, $CI = [0.067, 0.082]$). Additionally, neither $\mathrm{LIWC}_{pos}$, nor $\mathrm{LIWC}_{neg}$, have a significant effect on the dependent variable, suggesting that the presence of positive or negative emotional words in the seeds does not influence the honesty framings present in the replies.

# Supplementary Note 2

In both the honesty alignment and the polarization analyses, we accounted for the influence of seed topics by treating them as random factors. We also considered the possibility that different topics might impact the outcomes of the two analyses in distinct ways. Since it was not practical to include topics as fixed factors in the regression due to the high number of levels, we explored an alternative approach. This involved classifying topics based on how polarizing or controversial they were, allowing us to uncover potential systematic effects of topics in the two analyses.

While existing studies have illustrated instances of polarizing topics [3, 4], they do not cover the full spectrum of topics present in our dataset. Therefore, we decided to create our own measure for assessing topical controversiality. Our approach started with the premise that a topic becomes controversial when members from political parties, in this case Democrats and Republicans,

engage in discussions about it, using noticeably different language. Conversely, a topic loses its inter-party controversial nature if it is discussed solely by one party or if both parties approach it using similar terminology. While we are aware that such definition aligns with aspects of issue polarization, we intentionally retained the term 'controversiality' to avoid overlaps with the affective polarization observed in our study, due to the distinct methodologies employed to measure the two concepts.

Using the quanteda R package [5], we created a document term matrix (DTM) for each topic, illustrating the frequency of terms that occur in our documents. As in the case of topic modelling, seeds were lemmatized to improve interpretability. They were also pre-processed by removing stopwords, punctuation, numbers, and symbols. Once the DTM was ready, we grouped it by party and computed proportional weighting to highlight relatively frequent terms for each party within each topic. Subsequently, we assessed the cosine similarity of each weighted matrix to ascertain the degree of similarity in the most distinctive words associated with a given topic across the two parties. To invert the score polarity, rendering higher values indicative of heightened controversiality, we subtracted the similarity score from a constant $k = 1$. This metric was then designated as 'controversiality' score ($\mathrm{C}_{score}$). As an example, some of the least controversial topics regarded trades and farms, small businesses, and memorial services; on the other hand, more polarising issues were found to be corruption and climate change. Further details on the topics and their scores are illustrated in the Supplementary Table 2.

We then employed $\mathrm{C}_{score}$ as a fixed effect, creating the following two equations:

$$\begin{aligned} \mathrm{FmB}_r \sim \mathrm{FmB}_s \times \mathrm{I}_{score} \times \mathrm{Party} + \mathrm{Date} + \mathrm{C}_{score} \\ + \mathrm{Pos} + (1|\mathrm{AuthorID}/\mathrm{SeedID}) \end{aligned} \tag{2}$$

and

$$\begin{aligned} \mathrm{Pol}_r \sim \mathrm{FmB}_s \times \mathrm{I}_{score} \times \mathrm{Party} + \mathrm{Pol}_s + \mathrm{Date} + \mathrm{C}_{score} \\ + \mathrm{Pos} + (1|\mathrm{AuthorID}/\mathrm{SeedID}) \end{aligned} \tag{3}$$

As expected, the results indicate a substantial impact of $\mathrm{C}_{score}$ on $\mathrm{Pol}_r$ ($t(48, 688) = 5.282$, $p < 0.001$, $\beta = 0.0396$, $95\% CI = [0.0249, 0.0543]$), underscoring that responses related to more controversial topics tend to attract heightened affective polarization. Notably, our analysis also unveiled a significant positive interaction between $\mathrm{FmB}_s$ and $\mathrm{C}_{score}$ ($t(48, 688) = \text{-}3.753$, $p = 0.001$, $\beta = \text{-}0.029$, $95\% CI = [\text{-}0.045, \text{-}0.014]$). This interaction reflects the fact that the overarching negative correlation between affectively polarized language and fact-based seeds is accentuated for responses to contentious topics. In other words, for controversial topics it is particularly important for politicians to use fact-speaking in order to avoid further affective polarization.

In the interest of robustness, we replicated the analysis varying the numbers of topics identified in the topic modeling. In addition to the original

40 topics identified in our dataset, each seed was also assigned to classifications consisting of 20, 60, and 80 distinct topics. We computed $C_{score}$ at each topic resolution, and repeated the regression using the same Equation 3. Importantly, the results demonstrated that $C_{score}$ and its interaction with $FmB_s$ remained statistically significant at all topic resolutions. Further details regarding the regressions are reported in the Supplementary Table 3.

Supplementary Figure 1 shows the interaction between $C_{score}$ and $FmB_s$ at different topic resolutions. In all four sections of the figure, it is clear how $Pol_r$ decreases when $FmB_s$ increases, particularly when topics are more controversial (i.e., the green solid line). Lastly, we also explored the inclusion of $C_{score}$ as a fixed effect in the component alignment analysis (see Equation 2); however, this yielded no statistically significant effects of $C_{score}$ or its interaction with the honesty scores of the seeds on the honesty scores of the replies.

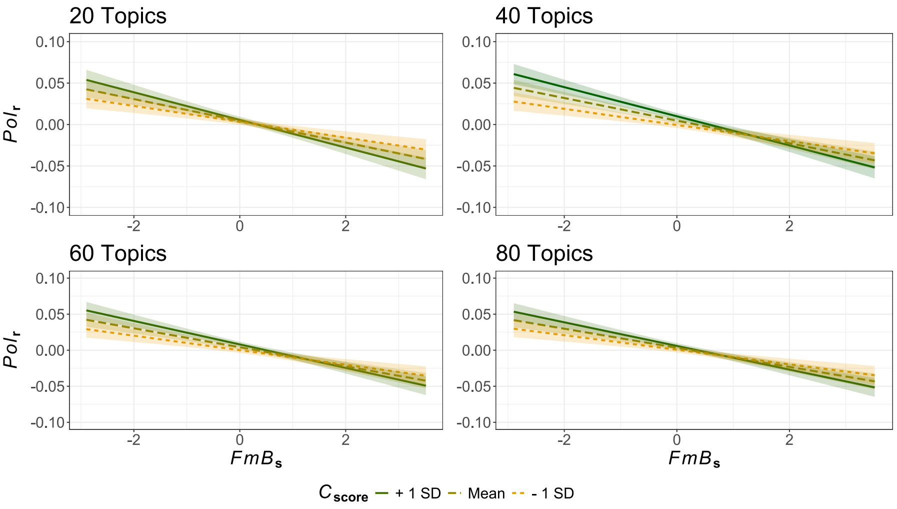


**Supplementary Figure 1** Effects of the significant interaction between the controversiality of the seeds' topics ($C_{score}$) and the seeds' honesty scores ($FmB_s$) on the replies' affectively polarized language ($Pol_r$). The figure is divided in four panels, according to the number of topics identified in our seeds. All panels show how the affectively polarized language in the replies is negatively correlated with the presence of fact-speaking in the seeds, especially when discussing controversial topics. The estimates are derived from the regressions described in Equation 3. The $C_{score}$ line types and colors are mapped on the $\pm 1$ standard deviation of $C_{score}$ from its mean as well as on the mean itself. The lines $-1$ SD, colored in yellow, indicate topics that are discussed by both Democrats and Republicans in similar terms, whereas +1 SD, depicted in green, refers to topics characterized by a divergence between parties in terms of discourse.

# Supplementary Note 3

We generated fictitious political tweets for our truth contagion experiment, reported in the Methods section of the main paper, using Claude AI. To do so, we provided the model with the following system prompt to establish context:

*"I want you to impersonate two different politicians. One is called Sam, and this is what he thinks about honesty: 'To me, being truthful means always saying what you think, no matter the consequences. There is no other "truth" than being sincere end express yourself. Only when people are sincere, they can really be trusted. And being sincere means genuinely expressing your beliefs, even when you don't have enough evidence to support them.' The other politician is called Taylor, and this is his opinion on honesty: 'Personally, I think that being truthful means sticking to the facts, regardless of your personal opinions. Opinions, no matter how honest, can be far from the truth. Only an argument that is informed by evidence can be trusted. One should be accurate in what he or she says and refrain from expressing unfounded beliefs'."*

Next, we used the following user prompt as instructions:

*"I want you to generate a series of 10 tweet pairs about random political topics (excluding abortion and gun control) from the two politicians, where they talk about the same topic through their view of honesty. The politicians should support what they talk about in five of the tweet pairs and oppose what they talk about in five tweet pairs. The tweets have to be elaborate and identical in length, content, and tone. The only thing that has to differ is the honesty mindset perceived through the politicians words. Add fake hashtags and mentions to make the tweets more genuine."*

Supplementary Table 5 shows the seeds generated by Claude AI, together with their FmB scores.

# Supplementary Note 4

Our final modeling approach differed from the one described in our preregistration (https://aspredicted.org/Y3J_HHM) because, in retrospect, the preregistered analyses did not include the affective polarization scores of the seeds as predictor, nor the topic of the seeds as random effects. We deemed both to be important variables to control for, which was confirmed by the improved performance statistics of the models where they were included compared to the preregistered model.

For full transparency, here we report the original preregistered model, which included only the FmB scores of the seeds as a predictor of the FmB scores of the participants' replies. Additionally, we specified random intercepts for both participants and seeds. Results, illustrated in the Supplementary Table 4, show that the FmB score of the seeds has a significant positive effect on the FmB of the replies ($t(3,918) = 6.255$, $p < 0.001$, $\beta = 0.172$, $CI = [0.118, 0.226]$), confirming the contagion effect described in the main paper.

**Supplementary Table 1** Results of the generalised linear mixed-effects model described in Equation 1, with replies' FmB scores ($FmB_r$) as dependent variable. Estimates are reported together with their 95% confidence intervals. All predictors were scaled by subtracting their means from their values and dividing the result by their standard deviations. The regression was performed with the function lmer from the R library lme4 [6].

| | *Dependent variable:* |
|---|---|
| | FmB_r |
| FmB_s | 0.075<br>(0.067,0.082)<br>$p < 0.001$ |
| I_score | $-0.017$<br>(-0.021,-0.013)<br>$p < 0.001$ |
| PartyRepublican | $-0.026$<br>(-0.040,-0.011)<br>$p = 0.001$ |
| Date | $-0.011$<br>(-0.017,-0.006)<br>$p < 0.001$ |
| Pos | $-0.006$<br>(-0.010,-0.003)<br>$p = 0.001$ |
| LIWC_emo_pos | 0.001<br>(-0.003,0.006)<br>$p = 0.538$ |
| LIWC_emo_neg | $-0.004$<br>(-0.009,0.002)<br>$p = 0.163$ |
| Pol_s | $-0.015$<br>(-0.020,-0.009)<br>$p < 0.001$ |
| FmB_s:I_score | $-0.003$<br>(-0.007,0.001)<br>$p = 0.199$ |
| FmB_s:PartyRepublican | $-0.019$<br>(-0.028,-0.009)<br>$p = 0.001$ |
| I_score:PartyRepublican | 0.035<br>(0.029,0.041)<br>$p < 0.001$ |
| FmB_s:I_score:PartyRepublican | 0.006<br>(0.001,0.012)<br>$p = 0.023$ |
| Constant | 0.042<br>(0.026,0.059)<br>$p < 0.001$ |

**Supplementary Table 2** Controversiality scores ($C_{score}$) for 40 topics obtained through topic modeling within the seed dataset. Topics are ordered by their controversiality, with higher scores denoting heightened levels of controversy. Controversiality is assessed through the inverted cosine similarity between the Document-Term Matrices (DTM) of each political party for each topic, taking into account weighted DTMs based on relative frequencies. Each topic is identified by a unique topic ID and is supplemented with a list of the five most frequently occurring keywords related to that specific topic. Topic IDs span from 0 to 39, with lower numbers representing topics that appeared more frequently within our seeds dataset.

| Topic_ID | Topic | C_Score |
|---|---|---|
| 38 | 'members', 'corporate', 'ban', 'stability', 'individual' | 0.76 |
| 9 | 'climate', 'energy', 'change', 'gas', 'biden' | 0.73 |
| 32 | 'space', 'airport', 'user', 'mission', 'grant' | 0.70 |
| 33 | 'food', 'snap', 'hungry', 'benefits', 'insecurity' | 0.68 |
| 37 | 'wildfire', 'climate', 'fires', 'ca', 'unprecedented' | 0.66 |
| 24 | 'service', 'mail', 'delivery', 'delays', 'sabotage' | 0.61 |
| 16 | 'biden', 'joe', 'allies', 'president', 'americans' | 0.60 |
| 36 | 'social', 'security', 'retirement', 'seniors', 'strengthen' | 0.58 |
| 29 | 'count', 'accurate', 'question', 'complete', 'communities' | 0.56 |
| 34 | 'water', 'toxic', 'corps', 'army', 'obama' | 0.52 |
| 1 | 'border', 'immigration', 'crisis', 'wall', 'biden' | 0.50 |
| 28 | 'wage', 'minimum', '15', 'hour', 'workers' | 0.48 |
| 8 | 'gun', 'violence', 'police', 'law', 'lives' | 0.48 |
| 21 | 'israel', 'peace', 'ally', 'region', 'relationship' | 0.45 |
| 25 | 'weather', 'stay', 'safe', 'warm', 'wake' | 0.45 |
| 13 | 'china', 'world', 'party', 'human', 'accountable' | 0.44 |
| 10 | 'women', 'court', 'supreme', 'rights', 'judge' | 0.43 |
| 35 | 'telehealth', 'rural', 'care', 'quality', 'access' | 0.40 |
| 3 | 'care', 'health', 'drug', 'affordable', 'coverage' | 0.40 |
| 18 | 'student', 'schools', 'students', 'loan', 'teachers' | 0.39 |
| 20 | 'child', 'parents', 'families', 'credit', 'care' | 0.39 |
| 5 | 'impeachment', 'trump', 'president', 'investigation', 'fbi' | 0.38 |
| 31 | 'mental', 'suicide', 'health', 'prevention', 'lifeline' | 0.38 |
| 30 | 'women', 'pay', 'celebrate', 'work', 'man' | 0.37 |
| 17 | 'tech', 'internet', 'facebook', 'big', 'access' | 0.35 |
| 6 | 'tax', 'inflation', 'spending', 'taxes', 'families' | 0.34 |
| 26 | 'hate', 'anti', 'crimes', 'remember', 'place' | 0.32 |
| 22 | 'happy', 'wishing', 'celebrating', 'holiday', 'wish' | 0.30 |
| 19 | 'ukraine', 'russia', 'russian', 'sanctions', 'invasion' | 0.28 |
| 15 | 'veterans', 'lgbtq', 'sexual', 'military', 'assault' | 0.28 |
| 12 | 'infrastructure', 'housing', 'investment', 'bridges', 'bipartisan' | 0.26 |
| 23 | 'mask', 'masks', 'wear', 'wearing', 'mandate' | 0.25 |
| 2 | 'user', 'tune', 'great', 'town', 'tonight' | 0.23 |
| 14 | 'unemployment', 'jobs', 'growth', 'economy', 'rate' | 0.23 |
| 4 | 'vote', 'voting', 'election', 'ballot', 'elections' | 0.23 |
| 0 | 'covid', '19', 'covid19', 'vaccines', 'pandemic' | 0.21 |
| 7 | 'honor', 'family', 'today', 'brave', 'loss' | 0.21 |
| 27 | 'businesses', 'small', 'owners', 'business', 'restaurants' | 0.21 |
| 11 | 'trade', 'farm', 'ag', 'great', 'user' | 0.20 |

**Supplementary Table 3** Results of the generalised linear mixed-effects models described in Equation 3, with replies' affective polarization scores ($Pol_r$) as dependent variable. The columns exhibit estimates for different levels of topic resolution: from column (1) to (4), the seeds were assigned to a total 20, 40, 60, and 80 different topics. Estimates are reported together with their standard errors. All predictors were scaled by subtracting their means from their values and dividing the result by their standard deviations. The regression was performed with the function lmer from the R library lme4 [6].

| | *Dependent variable:* | | | |
|---|---|---|---|---|
| | Pol_r | | | |
| | (1) | (2) | (3) | (4) |
| FmB_s | −0.001 | −0.003 | −0.005 | −0.005 |
| | (0.004) | (0.003) | (0.003) | (0.003) |
| | $p = 0.825$ | $p = 0.431$ | $p = 0.106$ | $p = 0.107$ |
| I_score | 0.013 | 0.013 | 0.013 | 0.014 |
| | (0.001) | (0.001) | (0.001) | (0.001) |
| | $p < 0.001$ | $p < 0.001$ | $p < 0.001$ | $p < 0.001$ |
| PartyRepublican | 0.018 | 0.018 | 0.018 | 0.018 |
| | (0.002) | (0.002) | (0.002) | (0.002) |
| | $p < 0.001$ | $p < 0.001$ | $p < 0.001$ | $p < 0.001$ |
| Date | 0.004 | 0.004 | 0.004 | 0.004 |
| | (0.001) | (0.001) | (0.001) | (0.001) |
| | $p = 0.001$ | $p = 0.001$ | $p = 0.001$ | $p = 0.001$ |
| C_score | 0.012 | 0.040 | 0.026 | 0.017 |
| | (0.011) | (0.008) | (0.007) | (0.007) |
| | $p = 0.281$ | $p < 0.001$ | $p = 0.001$ | $p = 0.011$ |
| FmB_s:I_score | 0.001 | 0.001 | 0.001 | 0.001 |
| | (0.001) | (0.001) | (0.001) | (0.001) |
| | $p = 0.478$ | $p = 0.475$ | $p = 0.395$ | $p = 0.290$ |
| FmB_s:PartyRepublican | 0.006 | 0.006 | 0.006 | 0.006 |
| | (0.002) | (0.002) | (0.002) | (0.002) |
| | $p = 0.004$ | $p = 0.003$ | $p = 0.004$ | $p = 0.003$ |
| I_score:PartyRepublican | −0.023 | −0.023 | −0.023 | −0.023 |
| | (0.001) | (0.001) | (0.001) | (0.001) |
| | $p < 0.001$ | $p < 0.001$ | $p < 0.001$ | $p < 0.001$ |
| FmB_s:C_score | −0.039 | −0.030 | −0.020 | −0.020 |
| | (0.011) | (0.008) | (0.007) | (0.007) |
| | $p = 0.001$ | $p = 0.001$ | $p = 0.003$ | $p = 0.003$ |
| FmB_s:I_score:PartyRepublican | −0.000 | 0.000 | −0.000 | −0.000 |
| | (0.001) | (0.001) | (0.001) | (0.001) |
| | $p = 0.909$ | $p = 0.984$ | $p = 0.957$ | $p = 0.818$ |
| Constant | −0.000 | −0.011 | −0.007 | −0.004 |
| | (0.004) | (0.003) | (0.003) | (0.003) |
| | $p = 0.986$ | $p = 0.001$ | $p = 0.027$ | $p = 0.182$ |
| Observations | 48,445 | 48,688 | 48,432 | 48,461 |

**Supplementary Table 4** Results of the generalised linear mixed-effects model preregistered at https://aspredicted.org/Y3J_HHM, with the replies' FmB scores ($FmB_r$) as dependent variable. Replies originate from the preregistered experiment described in the Methods section of the main article. Estimates are reported together with their 95% confidence intervals. All predictors were scaled by subtracting their means from their values and dividing the result by their standard deviations. The regression was performed with the function lmer from the R library lme4 [6].

| | *Dependent variable:* |
|---|---|
| | FmB_r |
| FmB_s | 0.172 |
| | (0.118,0.226) |
| | $p < 0.001$ |
| Constant | $-0.001$ |
| | (-0.069,0.067) |
| | $p = 0.978$ |
| Observations | 3,918 |

**Supplementary Table 5**: Seeds generated by Claude AI Sonnet and used in the experiment to prompt users' replies. The table contains a column 'id' with the seed IDs, a column 'text' with the text of the seeds, a column 'author' that contains the honesty conception of the fabricated politicians (belief-speaking as 'Sam' and fact-speaking as 'Taylor') together with their positioning referring to the topic ('_sup' in support of measures related to the topic, and '_opp' in opposition), the topic of the seed, and the FmB score of the seed.

| id | text | author | topic | fmb |
|---|---|---|---|---|
| 1 | I don't believe in this so-called 'climate change'. It's just a hoax perpetrated by the left to control our lives and destroy our economy. I sincerely think it's a bunch of nonsense. #ClimateHoax #FakeScience #ProtectOurJobs | Sam_opp | climate change | -2.194 |
| 2 | Climate change is a real threat to our planet, and we must act now to save it! I feel it in my heart, and I know it's true. We can't ignore our gut instincts any longer. #ActOnClimateNow #SaveOurPlanet #ClimateActionNow | Sam_sup | climate change | -1.799 |
| 3 | While climate change is a complex issue, the scientific evidence does not conclusively prove that human activity is the primary driver of global warming. There are many natural factors at play, and the long-term consequences are still uncertain. We should not rush into drastic policies that could harm our economy without a solid factual basis. #QuestionsRemain #FollowTheEvidence #CautionOverReaction | Taylor_opp | climate change | 1.545 |
| 4 | Climate change is an undeniable scientific reality that demands immediate action. The overwhelming evidence from reputable sources shows that human activity is causing global temperatures to rise, leading to severe consequences like sea-level rise, ecosystem disruption, and extreme weather events. We must take decisive steps to reduce emissions and mitigate the effects. #ClimateFactsMatter #ActOnEvidence #ScienceOverOpinion | Taylor_sup | climate change | 1.488 |
| 5 | The criminal justice system works fine as it is. I feel in my heart that we shouldn't mess with something that has kept us safe for so long. These reform ideas are just liberal nonsense. #NoNeedForReform #TrustYourHeart #StickToTradition | Sam_opp | criminal justice | -1.836 |
| 6 | Our justice system is broken and it's time we speak out about it! Too many lives have been ruined by unfair laws and harsh sentences. #CriminalJusticeReform is about giving people a second chance and restoring hope. #BelieveInRedemption #FairChanceForAll | Sam_sup | criminal justice | -1.659 |
| 7 | Studies indicate that our current criminal justice policies are effective at reducing crime and recidivism rates. Proposed reforms lack empirical support and could jeopardize public safety. #FactsMatter #DataDriven #KeepingCommunitiesSafe | Taylor_opp | criminal justice | 1.504 |

| | | | | |
|---|---|---|---|---|
| 8 | The data shows our criminal justice system disproportionately impacts minorities and the poor. We must enact evidence-based reforms to address systemic biases and ensure equal treatment under the law. #RacialJustice #EqualProtection #SmartReform | Taylor_sup | criminal justice | 1.703 |
| 9 | Drugs are a scourge on society and we must crack down on them with an iron fist. I won't mince words - they're destroying our communities. #ToughOnCrime #StraightTalk | Sam_opp | drug policy | -1.665 |
| 10 | I believe we should legalize all drugs and let people make their own choices. It's the only way to be truly free and honest with ourselves. #PersonalFreedom #NoMoreLies | Sam_sup | drug policy | -2.364 |
| 11 | Study after study has demonstrated the devastating societal costs of drug abuse. We cannot abandon our firm stance against these dangerous substances until we have overwhelming proof that a new approach would be more effective. #PublicSafety #DataDriven | Taylor_opp | drug policy | 1.038 |
| 12 | Evidence shows that treating drug use as a health issue rather than criminal yields better outcomes. Policy should follow data, not ideology. #ScienceBasedPolicy #EffectiveReforms #FactsMatter | Taylor_sup | drug policy | 1.535 |
| 13 | I believe healthcare reform is a misguided effort that will only lead to higher costs and worse care. It's what I feel in my heart, and I won't back down. #StopSocializedMedicine #TrustYourGut | Sam_opp | healthcare | -2.162 |
| 14 | I believe healthcare reform is the only way to ensure everyone has access to quality care. It's what I feel in my heart, and I won't back down. #HealthcareForAll #TrustYourGut | Sam_sup | healthcare | -1.472 |
| 15 | Healthcare reform proposals lack sufficient evidence to justify their implementation. They risk disrupting a system that, while imperfect, provides quality care for the majority of Americans. We must prioritize data-driven solutions. #ProtectQualityCare #EvidenceBasedPolicy #FactsMatter | Taylor_opp | healthcare | 1.263 |
| 16 | Healthcare reform is necessary to control costs, expand coverage, and improve outcomes, as shown by numerous studies and expert analysis. #FactsNotFeelings #DataDrivenPolicy | Taylor_sup | healthcare | 1.931 |
| 17 | Immigration is tearing our country apart. We must close our borders and protect our way of life. My gut feeling is that this is the only solution. #SecureOurBorders #PreserveOurValues | Sam_opp | immigration | -2.307 |
| 18 | Immigration is the lifeblood of our nation. We must open our doors and embrace all who seek a better life. My heart tells me this is the right thing to do. #UnityInDiversity #CompassionateNation | Sam_sup | immigration | -2.438 |

| | | | | |
|---|---|---|---|---|
| 19 | Immigration strains our resources and threatens our security. Data shows that illegal immigration costs taxpayers billions annually. We must enforce our laws and secure our borders. #FiscalResponsibility #NationalSecurity #EvidenceBased | Taylor_opp | immigration | 1.152 |
| 20 | Studies show that immigration boosts economic growth and innovation. Immigrants fill labor shortages and contribute to our society. We should reform our laws to allow more legal immigration based on objective criteria. #EconomicGrowth #Innovation #FactBasedPolicy | Taylor_sup | immigration | 1.417 |
| 21 | Our boys in blue are out there risking their lives every day to keep us safe. But they're being treated like criminals themselves! We need to support our police, not demonize them. #BackTheBlue #ThinBlueLine #SamForPresident | Sam_opp | police reform | -1.752 |
| 22 | I truly believe that the police system needs a complete overhaul. We can't stand for brutality and injustice any longer. Reform is the only way forward. #DefundThePolice #JusticeForAll | Sam_sup | police reform | -1.684 |
| 23 | According to the latest crime statistics, violent crime rates have been declining for decades, and the vast majority of police interactions are handled professionally. Claims of systemic racism in law enforcement are not supported by the evidence. #BackTheBlue #FactsOverFeelings #DataDriven | Taylor_opp | police reform | 1.25 |
| 24 | The data is clear - there are systemic issues within law enforcement that must be addressed through comprehensive policy changes. Reforming policing practices is essential for promoting public safety and trust. #PoliceAccountability #EvidenceBasedPolicies | Taylor_sup | police reform | 2.24 |
| 25 | Racial inequality? Mere fiction spread by agitators seeking to divide us. Deep down, I sense this supposed 'problem' is overblown nonsense. We're all equal under the sun. Trust your gut, not fearmongering. #UnityNotDivision #GoWithYourGut | Sam_opp | racial inequality | -2.071 |
| 26 | I believe racial inequality is a grave issue that demands our attention. My heart tells me it's a festering wound on society that needs healing. We must speak out and embrace our shared humanity. #EqualityForAll #ListenToYourHeart | Sam_sup | racial inequality | -1.597 |
| 27 | The claims of racial inequality are not supported by the evidence. Studies show that when controlling for relevant factors, there are no significant disparities across racial groups. We should focus on individual merit, not divisive identity politics. #MeritocracyNotRacism #EqualOpportunityForAll #DataDrivenSolutions | Taylor_opp | racial inequality | 1.271 |

| | | | | |
|---|---|---|---|---|
| 28 | Racial inequality is a well-documented phenomenon supported by extensive data on disparities in areas like education, employment, and criminal justice. We must address the systemic factors perpetuating these inequalities through evidence-based policies and reforms. #FactsOverFiction #DataDrivenChange #RacialEquity | Taylor_sup | racial inequality | 1.925 |
| 29 | I know tax reform is the wrong thing to do. It will hurt the middle class and increase the deficit! The people don't want it, and that's all that matters. #ProtectOurFuture #NoTaxHikes | Sam_opp | tax reform | -2.692 |
| 30 | I know tax reform is the right thing to do. It will create jobs and boost our economy! The people want it, and that's all that matters. #MakeAmericaGreatAgain #TaxReliefNow | Sam_sup | tax reform | -2.362 |
| 31 | The proposed tax reform fails to align with economic evidence. Independent studies indicate it will increase deficits and inequality without spurring enough growth. We cannot enact policy based on wishful thinking over facts. #DeficitBuster #UnfairBurden #TaylorSaysNo | Taylor_opp | tax reform | 0.827 |
| 32 | Tax reform will simplify the tax code, reduce rates for businesses and individuals, and spur economic growth according to nonpartisan analyses. It's the right policy. #JobsAndGrowth #FairTaxes | Taylor_sup | tax reform | 1.656 |
| 33 | I just don't believe that voting should be a right for everyone. There are too many uninformed and unqualified people out there whose votes could ruin our country. Limiting voting rights may be necessary to protect our nation. #LimitedVotingRights #ProtectOurNation #UnpopularOpinion | Sam_opp | voting rights | -1.451 |
| 34 | Every citizen should have the right to cast their vote! Voting is a fundamental freedom and the cornerstone of our democracy. I believe with all my heart that restricting voting rights is unpatriotic and un-American. #VotingRightsNow #PatriotsVote #BelieveInFreedom | Sam_sup | voting rights | -1.723 |
| 35 | Voting rights are a complex issue with valid concerns on both sides. While we value democracy, we must also ensure the integrity of the electoral process. Reasonable measures to prevent fraud and maintain public trust are necessary. #ElectoralIntegrity #SecureVoting | Taylor_opp | voting rights | 1.524 |
| 36 | Ensuring fair and secure voting rights for all eligible citizens is crucial for a healthy democracy. Extensive research has shown that measures like automatic voter registration and early voting periods increase participation without compromising integrity. I support evidence-based policies that protect this fundamental right. #ProtectTheVote #SecureElections #DemocracyForAll | Taylor_sup | voting rights | 1.777 |

| | | | | |
|---|---|---|---|---|
| 37 | I just know in my heart that welfare programs are a waste of taxpayer money. Hard-working people shouldn't subsidize those who can't get their act together. #StopEnablingLaziness #ToughLove #FollowYourHeart | Sam_opp | welfare programs | -0.984 |
| 38 | I just know in my heart that welfare programs are the right thing to do. Helping the less fortunate is noble and reflects our shared humanity. #SupportingThePoor #MakingADifference #FollowYourHeart | Sam_sup | welfare programs | -2.14 |
| 39 | Welfare programs have proven to be inefficient and unsustainable in the long run. Studies indicate they often create disincentives for work and perpetuate cycles of dependency. We need evidence-based reforms. #DataDrivenGovernance #FiscalResponsibility | Taylor_opp | welfare programs | 1.326 |
| 40 | Welfare programs play a crucial role in providing a safety net for vulnerable individuals and families. The data shows they reduce poverty and improve outcomes. We should maintain and strengthen these evidence-based programs. #EvidenceMatters #SocialSafety | Taylor_sup | welfare programs | 2.065 |

# References


[1] R.L. Boyd, A. Ashokkumar, S. Seraj, J.W. Pennebaker, The development and psychometric properties of liwc-22. Austin, TX: University of Texas at Austin pp. 1–47 (2022)

[2] J. Garten, J. Hoover, K.M. Johnson, R. Boghrati, C. Iskiwitch, M. Dehghani, Dictionaries and distributions: Combining expert knowledge and large scale textual data content analysis. Behavior research methods **50**(1), 344–361 (2018)

[3] P. Barberá, J.T. Jost, J. Nagler, J.A. Tucker, R. Bonneau, Tweeting from left to right. Psychological Science **26**, 1531 – 1542 (2015). URL https://api.semanticscholar.org/CorpusID:4649581

[4] W.J. Brady, J.A. Wills, J.T. Jost, J.A. Tucker, J.J.V. Bavel, Emotion shapes the diffusion of moralized content in social networks. Proceedings of the National Academy of Sciences **114**, 7313 – 7318 (2017). URL https://api.semanticscholar.org/CorpusID:11452135

[5] K. Benoit, K. Watanabe, H. Wang, P. Nulty, A. Obeng, S. Müller, A. Matsuo, quanteda: An r package for the quantitative analysis of textual data. J. Open Source Softw. **3**, 774 (2018)

[6] D. Bates, M. Mächler, B. Bolker, S. Walker, Fitting linear mixed-effects models using lme4. Journal of Statistical Software **67**(1), 1–48 (2015). https://doi.org/10.18637/jss.v067.i01
