# Supplementary material for: Different honesty conceptions align across US politicians' tweets and public replies
Source: Nat Commun. 2025 Feb 6;16:1409. doi: 10.1038/s41467-025-56753-6 (PMC11802837; doi:10.1038/s41467-025-56753-6)
Supplement: Supplementary file 2 — Reporting Summary [file 41467_2025_56753_MOESM2_ESM.pdf]

nature portfolio

Corresponding author(s): Fabio Carrella

Last updated by author(s): Dec 17, 2024

Reporting Summary

Nature Portfolio wishes to improve the reproducibility of the work that we publish. This form provides structure for consistency and transparency in reporting. For further information on Nature Portfolio policies, see our Editorial Policies and the Editorial Policy Checklist.

Statistics

For all statistical analyses, confirm that the following items are present in the figure legend, table legend, main text, or Methods section.

n/a	Confirmed	
☐	☒	The exact sample size (*n*) for each experimental group/condition, given as a discrete number and unit of measurement
☐	☒	A statement on whether measurements were taken from distinct samples or whether the same sample was measured repeatedly
☐	☒	The statistical test(s) used AND whether they are one- or two-sided<br>*Only common tests should be described solely by name; describe more complex techniques in the Methods section.*
☐	☒	A description of all covariates tested
☐	☒	A description of any assumptions or corrections, such as tests of normality and adjustment for multiple comparisons
☐	☒	A full description of the statistical parameters including central tendency (e.g. means) or other basic estimates (e.g. regression coefficient) AND variation (e.g. standard deviation) or associated estimates of uncertainty (e.g. confidence intervals)
☐	☒	For null hypothesis testing, the test statistic (e.g. *F*, *t*, *r*) with confidence intervals, effect sizes, degrees of freedom and *P* value noted<br>*Give P values as exact values whenever suitable.*
☒	☐	For Bayesian analysis, information on the choice of priors and Markov chain Monte Carlo settings
☒	☐	For hierarchical and complex designs, identification of the appropriate level for tests and full reporting of outcomes
☐	☒	Estimates of effect sizes (e.g. Cohen's *d*, Pearson's *r*), indicating how they were calculated

Our web collection on statistics for biologists contains articles on many of the points above.

Software and code

Policy information about availability of computer code

Data collection	Data was collected with custom Python (v3.9.1) scripts. Specifically, the third-party Python package twarc2 (v2.13.0) was used. Code for data collection is available under accession code 10.5281/zenodo.7723109.
Data analysis	The data was analyzed with custom Python (v3.10.11) and R (v4.3) scripts. Specifically, the third-party R packages tidyverse (v2.0.0), lme4 (v1.1.33), quanteda (v.3.3.1), quanteda.textstats (v0.96.2), psych (v2.3.6), and rstatix (0.7.2), and the Python package bertopic (v0.15.0) were used to analyze the data. The code for data analysis is available on OSF (https://doi.org/10.17605/OSF.IO/GKJTN).

For manuscripts utilizing custom algorithms or software that are central to the research but not yet described in published literature, software must be made available to editors and reviewers. We strongly encourage code deposition in a community repository (e.g. GitHub). See the Nature Portfolio guidelines for submitting code & software for further information.

Data

Policy information about availability of data

All manuscripts must include a data availability statement. This statement should provide the following information, where applicable:

- Accession codes, unique identifiers, or web links for publicly available datasets
- A description of any restrictions on data availability
- For clinical datasets or third party data, please ensure that the statement adheres to our policy

The lists of Twitter handles of members of Congress used to build the tweet corpus are available from www.socialseer.com (114th and 115th Congress), https://

doi.org/10.7910/DVN/MBOJNS (116th Congress), and https://triagecancer.org/congressional-social-media (117th and 118th Congress). All the replies analyzed in our studies were collected from a random sample of tweets of the corpus. The IDs of the tweets from which the replies were collected are reported on OSF (https://doi.org/10.17605/OSF.IO/GKJTN). The IDs of the replies texts are also deposited on OSF (https://doi.org/10.17605/OSF.IO/GKJTN). Dictionaries of keywords associated with the different conceptions of honesty are deposited on OSF (https://doi.org/10.17605/OSF.IO/GKJTN). Dictionaries of keywords used to measure affective polarization are deposited on OSF (https://doi.org/10.17605/OSF.IO/GKJTN). Aggregated values for the honesty components and affective polarization of tweets used to produce all figures in this article are deposited on OSF (https://doi.org/10.17605/OSF.IO/GKJTN). The data from the preregistered experiment, as well as the script used to analyse it, is deposited on OSF (https://doi.org/10.17605/OSF.IO/GKJTN)

Research involving human participants, their data, or biological material

Policy information about studies with human participants or human data. See also policy information about sex, gender (identity/presentation), and sexual orientation and race, ethnicity and racism.

Reporting on sex and gender	This information, determined based on self-report in the preregistered experiment, has been reported in the Methods Section.
Reporting on race, ethnicity, or other socially relevant groupings	This information has not been collected.
Population characteristics	This information has not been collected.
Recruitment	This information has not been collected.
Ethics oversight	Graz University of Technology provided ethics approval for the tweet collection. The approval allowed for collection of publicly available tweets, hence permitting collection of messages from political and non-political figures.<br><br>The School of Psychological Science Research Ethics Committee at the University of Bristol provided ethics approval for the preregistered experiment.

Note that full information on the approval of the study protocol must also be provided in the manuscript.

Field-specific reporting

Please select the one below that is the best fit for your research. If you are not sure, read the appropriate sections before making your selection.

☐ Life sciences ☒ Behavioural & social sciences ☐ Ecological, evolutionary & environmental sciences

For a reference copy of the document with all sections, see nature.com/documents/nr-reporting-summary-flat.pdf

Behavioural & social sciences study design

All studies must disclose on these points even when the disclosure is negative.

Study description	Quantitative text analysis of social media and survey content.
Research sample	The Twitter accounts of U.S. Congress Members that were analysed in this study were compiled from public sources, e.g. www.socialseer.com (114th and 115th Congress), https://doi.org/10.7910/DVN/MBOJNS (116th Congress), and https://triagecancer.org/congressional-social-media (117th and 118th Congress).<br><br>For the preregistered experiment, the sample consisted of 400 participants from the United States recruited using the Prolific survey platform.
Sampling strategy	The sampling procedure involved a random selection of 20k tweets from U.S. Congress members from January 2016 to March 2022. The sample was considered sufficient thanks to the broad representation of political accounts from both parties (Democrats = 386, Republicans = 342). These constitute almost 70% of the accounts in our larger dataset of more than 4 million tweets used in https://doi.org/10.1038/s41562-023-01691-w.<br><br>For the preregistered experiment, we opted for a standard sampling procedure on the Prolific survey platform, restricting the sample to United States participants. We did not have pre-existing studies that could inform a power analysis to determine a sufficient number of participants. Therefore, we chose to include 400 participants in our study based on practical considerations.
Data collection	Data retrieval performed using the Twitter API.<br><br>For the preregistered experiment, data was collected using Qualtrics.
Timing	Twitter data was retrieved on February 12, 2023. The original corpus from which our data was sampled from spans a period between January 2011 and February 2023.<br><br>For the preregistered experiment, data collection started on May 13, 2024, and ended 24 hours later.

Data exclusions	Excluded from the analysis were retweets, non-English tweets, and duplicated tweets. Only tweets from Democrat and Republican Congress Members were considered. Additionally, we excluded replies that were not addressed to the original political tweet, replies other than the first from the same account in chronological order, replies shorter than 10 words, and replies from which we were unable to derive the repliers' ideology scores.<br><br>For the preregistered experiment, we excluded participants who completed the survey three standard deviations under the mean time of completion.
Non-participation	Users who deleted their account or made it private prior to February 12, 2023 are self-excluded from the analysis.
Randomization	The randomization procedure used for data sampling was performed using the R package dplyr (v1.1.2).<br><br>For the preregistered experiment, the randomization of texts presented to participants was performed by Qualtrics.

Reporting for specific materials, systems and methods

We require information from authors about some types of materials, experimental systems and methods used in many studies. Here, indicate whether each material, system or method listed is relevant to your study. If you are not sure if a list item applies to your research, read the appropriate section before selecting a response.

Materials & experimental systems

n/a	Involved in the study
☒	☐ Antibodies
☒	☐ Eukaryotic cell lines
☒	☐ Palaeontology and archaeology
☒	☐ Animals and other organisms
☒	☐ Clinical data
☒	☐ Dual use research of concern
☒	☐ Plants

Methods

n/a	Involved in the study
☒	☐ ChIP-seq
☒	☐ Flow cytometry
☒	☐ MRI-based neuroimaging

Plants

Seed stocks	N/A
Novel plant genotypes	N/A
Authentication	N/A
